# Alendronate (ALN) combined with Osteoprotegerin (OPG) significantly improves mechanical properties of long bone than the single use of ALN or OPG in the ovariectomized rats

**DOI:** 10.1186/1749-799X-6-34

**Published:** 2011-07-13

**Authors:** Yan Wang, Peng Huang, Pei-Fu Tang, Kai-Ming Chan, Gang Li

**Affiliations:** 1 Department of Orthopaedic Surgery, The General Hospital of People's Liberation Army, Beijing, PR China; 2 CUHK-Jockey Club Collaborating Centre for Sports Medicine and Health Sciences, The Chinese University Hong Kong, Prince of Wales Hospital, Shatin, Hong Kong, PR China; 3 Department of Orthopaedics & Traumatology and Stem Cells and Regenerative Medicine Laboratory, Li Ka Shing Institute of Heath Sciences, The Chinese University Hong Kong, Prince of Wales Hospital, Shatin, Hong Kong, PR China; 4 CUHK-Jian University Joint Laboratory, MOE Key Laboratory for Regenerative Medicine, School of Biomedical Sciences, The Chinese University of Hong Kong, Shatin, Hong Kong, PR China

**Keywords:** Osteoprotegerin, Alendronate, RANKL, Osteoporosis, Ovariectomy

## Abstract

**Background:**

Alendronate (ALN) is the most common form of bisphosphonates used for the treatment of osteoporosis. Osteoprotegerin (OPG) has also been shown to reduce osteoporotic changes in both humans and experimental animals after systemic administration. The aim of this current study was to test if the anti-resorption effects of ALN may be enhanced when used in combination with OPG.

**Objectives:**

To investigate the effects of ALN, OPG or combined on bone mass and bone mechanical properties in ovariectomized (OVX) rats.

**Methods:**

OVX rats were treated with ALN, OPG-Fc, or OPG-Fc and ALN. Biochemical markers, trabecular bone mass, biomechanics, histomorphometry and RANKL expression in the bone tissues were examined following the treatments.

**Results:**

The treatment of ALN, OPG-Fc and ALN+OPG-Fc all prevented bone loss in the OVX-rats, there was no statistical difference among the three treatment groups in terms of vertebrae BMD, mineralizing surfaces, mineral apposition rate, BFR/BS. The ALN+OPG-Fc treatment group had significantly increased the mechanical strength of lumber vertebral bodies and femoral shafts when compared to the ALN and OPG-Fc treatment groups. The RANKL protein expression in the vertebral bones was significantly decreased in the ALN and ALN+OPG-Fc treatment groups, suggesting the combined use of OPG-Fc and ALN might have amplified inhibition of bone resorption through inhibiting RANKL-dependent osteoclastogenesis.

**Conclusion:**

The combined use of OPG-Fc and ALN may be a new treatment strategy for reversing bone loss and restoring bone quality in osteoprotic disorders.

## Background

Receptor activator of the NF-κB ligand (RANKL), a key promoting factor for osteoclast differentiation, is expressed on osteoblastic cells. RANKL induces the differentiation/formation of osteoclasts by binding to RANK on the osteoclastic precursor cells. Clinical application of RANKL inhibition has a major effect on metabolic bone disease such as osteoporosis. Osteoprotegerin (OPG) inhibits RANKL-RANK pathway through competitive bindings to RANKL. OPG deficiency resulted in severe osteoporosis and systemic administration OPG can reduce osteoporotic changes in both humans and experimental animals [1]. However, relative large dose of OPG are needed for systemic administration which might cause undesired immune responses, and the high cost may prohibit OPG wider clinical applications.

Bisphosphonates are structurally analogous to pyrophosphate, having greater affinity to bone that can rapidly accumulate in bone tissue and induce osteoclast apoptosis through inhibiting farnesyl pyrophosphate (FPP) and GGPP biosynthesis and G-proteins. Alendronate (ALN) has been reported to inhibit GGPP biosynthesis in mevalonate pathway and the signal transduction in the Ras-mitogen-activated protein kinase pathway, thereby inhibiting RANKL expression [2]. Our in vitro data indicated that the combined use of ALN and OPG had greater inhibitory effect on osteoclastogenesis than the use of OPG and ALN alone [3]. At present there is no published data on the effect of combined use of OPG and ALN on osteoporostic bone loss *in vivo*. In the present study, we investigated the changes of biochemical markers, trabecular bone mass and bone biomechanics in ovariectomized (OVX) rats with ALN, OPG treatment alone or in combination.

## Methods

### Rat Ovariectomy Model and Experimental Groups

12 weeks old female Sprague-Dawley rats (Experimental Animal Center, Chinese General Hospital of PLA, Beijing, China), body weight 350-400 g were used for this experiment. The rats were maintained on commercial rat chow with 0.95% calcium and 0.67% phosphate. Rats were housed in a room that was maintained at 70°F with a 12-h light/dark cycle. All animals were treated according to the animal care guidelines, Department of Health, PR China with the approval of the PLA General Hospital Ethical Committee on Animal Research. Bilateral ovariectomies were performed using dorsal approach as previously reported.

**Figure 1:**
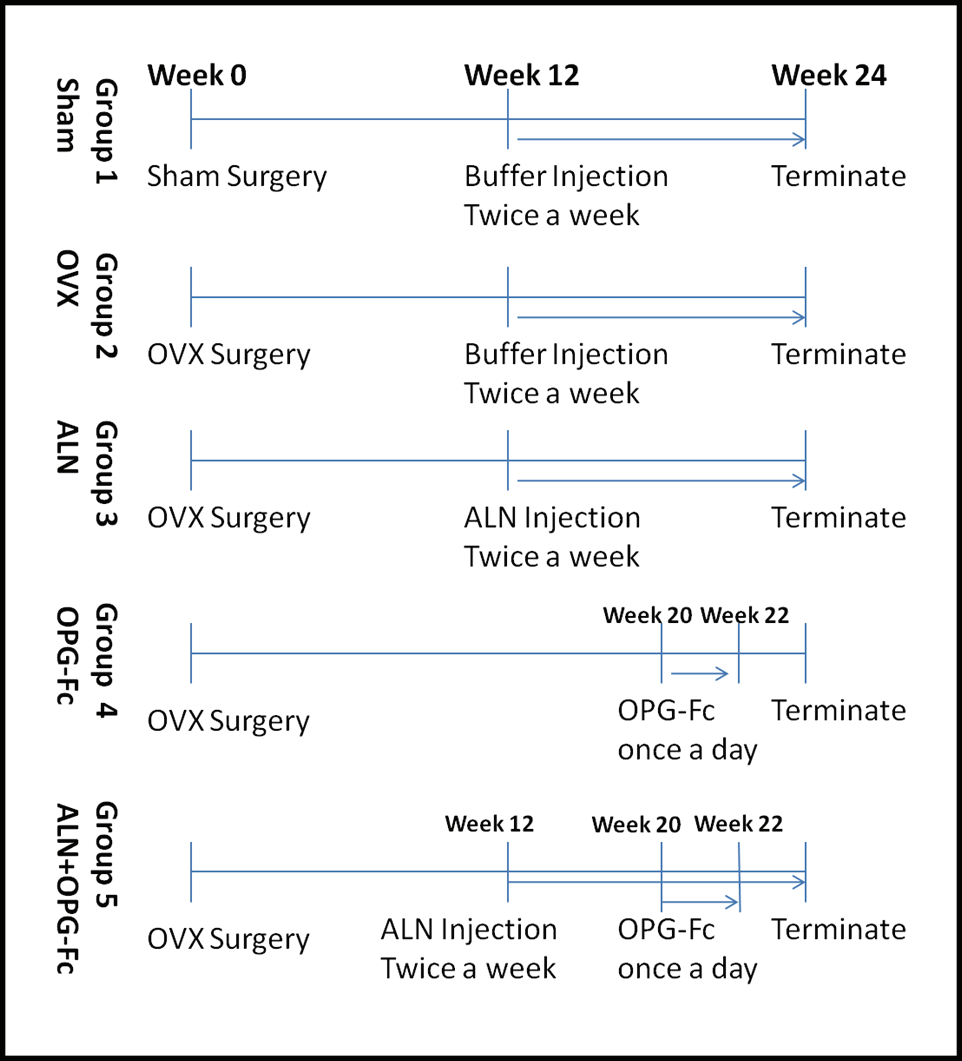
**Diagram shows the experimental details of treatment in each animal group**.

Fifty rats were divided into five groups with 10 animals in each group. Group 1: animals received sham surgery; ovaries were exteriorized but not removed. For Groups 2, 3, 4 and 5, all animals received bilateral ovariectomies and waited for 12 weeks, and then used in the following experiments (as detailed in Figure 1): Group 1 (Sham) and Group 2 (OVX) received subcutaneous injection of the vehicle buffer twice weekly for 12 weeks. The other 3 groups were treated as the following: Group 3, OVX + ALN, 28 μg/kg subcutaneous injection twice/week for 12 weeks; Group 4, OVX + rhOPG-Fc, animals received 5 mg/kg rhOPG-Fc subcutaneous injection per day for 2 weeks starting from 20 weeks after the OVX surgery; Group 5, OVX + rhOPG-Fc+ ALN, the animals were treated twice/week with 28 μg/kg ALN subcutaneous injection, and 5 mg/kg rhOPG-Fc was injected daily for 2 weeks starting from 20 weeks after the OVX surgery. All animals were killed at 24 weeks following OVX surgery and bone samples collected for further examinations.

### Materials

Anti-RANKL antibody was purchased from Santa Cruz, USA. BCA Protein assay kit was from PIERCE, Rockford, IL, USA. Polyvinylidene difluoride membrane filter was from Millipore, Tokyo, Japan. The ECL system was from Amersham Biosciences, Co., Piscataway, NJ, USA. Densitometric analysis was done with an ATTO Densitograph (ATTO, Tokyo, Japan). ALN was obtained from Merck Company. Anti-β-actin mouse monoclonal antibody was from Sigma, USA. rhOPG-Fc (22-201 Amino acid) was from Fuchun Zhongnan Company, Shanghai, PR China and the details of the characterization of rhOPG-Fc was published previously [3].

### Bone Protein Preparation and Western Blotting

The 2^nd^-5^th ^caudal vertebrae were collected and prepared according to Miyazaki et al [4]. In brief, all connective tissues were removed and 100 mg of vertebral bone were crushed by surgical pliers, homogenized with BAP buffer (50 mM Tris-HCL buffer, pH 7.5, containing 0.3 mM phenylmethyl fluoride, 1.0 mM benzamidine, and 0.1% Triton X-100). The homogenate was centrifuged at 12,000 g for 20 min at 37°C. Resultant supernatant was used for Western blot analysis. Western blotting was performed using a previously described method [5]. β-actin was used as an internal standard; Equal amount of proteins separated by electrophoresis were transferred to 2 polyvinylidene difluoride membrane filter; the proteins on the membranes were incubated with antibodies of anti-RANKL and anti-β-actin overnight at 37°C. After washing, the filters were reacted with peroxidase-conjugated secondary antibodies for 60 minutes at room temperature. The reactive bands were detected with the ECL system, and the relative intensities of the bands were calculated and expressed by percentage change.

### Urine and Serum Biochemical Assays

Rats were housed in individual metabolic cages, fasted for 24 h before the urine samples were collected at the day before surgery, 12 weeks and 24 weeks days following OVX surgery. Twenty four-hour urine samples were collected over sodium azide in metabolic cages. To minimize contamination with dietary (soybean) proteins, the rats were starved during the collection period.

For serum collection, at each time-point, 500-1000 μl peripheral blood was collected from the tail vein with a plastic 1-ml heparinized syringe and further heparin (at a final concentration of approximately 0.5 mg heparin per ml of blood) was added to the blood. The red blood cells were removed by centrifugation for 10 min at 4500 × g and the serum were collected. The sera were filtered through a 0.22-μm filter for sterilization as well as in order to remove any fragments of platelets.

Urinary levels of calcium (Ca^2+^), phosphonate (P), creatinine (Cr) and serum levels of Ca, P were measured by standard laboratory tests. Serum alkaline phosphatase (ALP) and osteocalcin (OSC) were measured using ELISA plates from R&D system according to the manufacturer's instructions.

### Bone Mineral Density (BMD) Measurement

At day 0 (before surgery), 12 weeks (development of osteopenia), and 24 weeks (before killing) following OVX surgery, whole body BMD were measured using lunar-DXA IQ (Lunar company, USA) and the BMD at the 4^th^, 5^th ^and 6^th ^vertabrae. The subjects were placed on the scan table in the supine position. All scans were performed in slow mode and analyzed using Lunar smart scan version software with the slowest scan mode. The measurements of the hip and vertebrae were repeated three times, and the means were calculated.

### Biomechanical Testing

The mechanical properties reflect the true quality of bone and they are used as primary outcome measurement parameter in this study. Three-point bending test described by Turner and Bur [6] was used to measure the mechanical strength of intact femurs. The femur was placed on custom-made struts, 9 mm apart, with a 100-N superior load cell delivered through a superior strut directed to the mid-diaphyseal region at a rate of 1 mm/min. Load-displacement curves were recorded using a servo-hydraulic materials testing machine (858 Mini Bionix, MTS Corp., Minneapolis, MN, USA). Data were collected concerning peak load to failure, and stiffness was calculated from load-displacement curves, and elastic load, elastic stress and Young's modulus (maximum slope of the stress-strain curve) were calculated and compared. L3 vertebrae were also measured by vertical compression at the center along the cephalocaudal axis; the deformation and volume changes were determined. Biomechanical parameters including ultimate load (Fmax), maximum stress (Fmax/cross-sectional area), elastic load, elastic stress and Young's modulus (maximum slope of the stress-strain curve) were calculated and analyzed.

### Bone Histomorphometry

To determine bone formation rate, rats received subcutaneous injections of 10 mg/kg of Calcein at 12 and 2 days before termination. Tibiae were collected and embedded in methylmethacrylate. Serial longitudinal sections of 4-μm, 8-μm and 200-μm thickness were cut. 4-μm sections were stained with Von Kossa and toluidine blue; the 8-μm sections were mounted without staining for measuring the bone growth rate using the cacein labeling; the bone mineral apposition rate was demonstrated by the distance of the two calcein labeling lines divided by 10 days. The 200-μm thick sections were mounted without staining for taking digital photographs. Histomorphometry measurements were performed on the proximal metaphyseal region (between 2 and 4 mm distal to the growth plate/metaphyseal junction) using a digital image analysis system (Osteomeasure, Inc., Atlanta, GA, USA). Trabecular area, perimeter, single- and double-labeling surfaces, osteoclast number, osteoid surface were measured. Trabecular number, thickness, mineralizing surface, mineral appositional rate, BFR/surface volume (BFR/BS), and osteoclasts number per millimeter were calculated according to the methods reported by Parfitt et al [7].

### Statistics

The mean and standard deviation (SD) were shown. All the data were analyzed with one-way ANOVA test and the inter-relationship function of the two agents, ALN and OPG was analyzed using SPSS software (SPSS Version 10; SPSS Inc., Chicago, IL, USA). Significant difference was considered at p < 0.05.

## Results

### Effect of rhOPG-Fc and ALN Treatment on RANKL Protein Expression

**Figure 2:**
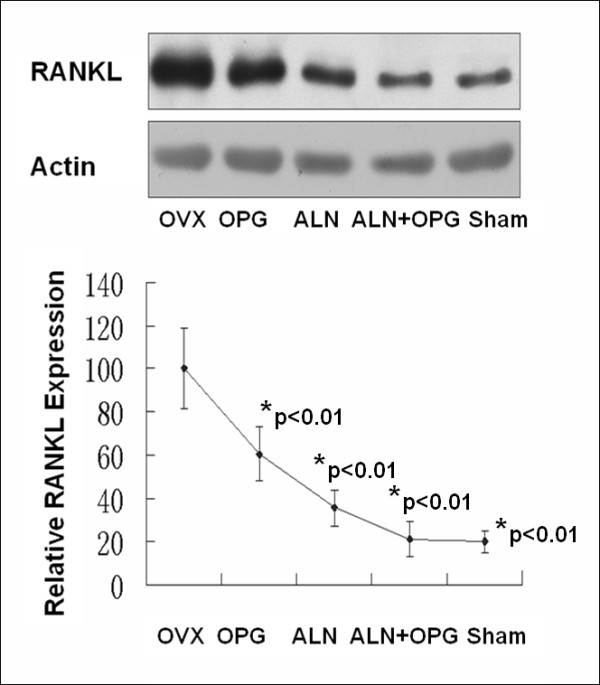
**Western blot analysis showed the RANKL protein expression in the vertebral bones at 12 weeks after receiving different treatments (24 weeks post-OVX)**. RANKL protein expression in the vertebrae was significantly increased in the OVX group compared to all other groups. If we use the RANKL protein expression in the OVX group as baseline level of 100%, the OPG-Fc treatment reduced the RANKL protein expression to 60% of baseline level; ALN treatment further reduced the RANKL protein expression to 40% of the baseline level; the RANKL protein expression in the OPG-Fc+ALN treated group and the sham control group was similar and was lowest among the groups, only 20% of the OVX group baseline level. *p < 0.05 when compared to the OVX group and there was no statistical difference among the tree treatment groups.

Western blot showed that RANKL protein expression in the vertebrae was significantly increased in the OVX group compared to all other groups (Figure 2, p < 0.01). If we use the RANKL protein expression in the OVX group as baseline level of 100%, the OPG-Fc treatment reduced the RANKL protein expression to 60% of baseline level; ALN treatment further reduced the RANKL protein expression to 40% of the baseline level; the RANKL protein expression in the OPG-Fc+ALN treated group and the sham control group was similar and the lowest among the groups, only 20% of the OVX group baseline level, however there is no statistical differences among the treatment groups (Figure 2).

### Changes of Biochemical Markers in Urine and Serum

**Figure 3:**
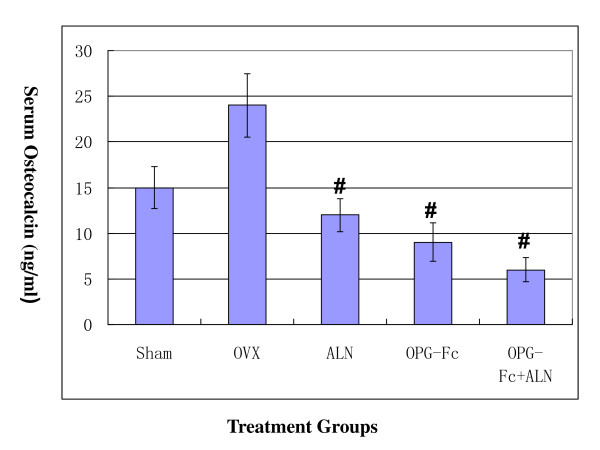
**At 24 weeks post OVX (12 weeks following treatment), serum osteocalcin level decreased by 23% to the baseline level (sham group) in the ALN-treated group; 35% to the baseline level in the OPG-Fc-treated group; and 58% to the baseline level in the OPG+ALN-treated group, which was significantly reduced (#p < 0.05, Student's t-test) comparing to the sham group**. There was no statistical difference among the three treatment groups.

The urinary excretion of calcium ion was similar in all groups at 12 weeks post OVX, but it was significantly increased in the OVX group at 24 weeks compare to other groups (Table 1). There was no significant change in urinary excretion of phosphates and creatinine (not shown). Serum osteocalcin level in the OVX group increased approximately 46% comparing to the sham group at 12 weeks post OVX and remained elevated at this level until 24 weeks post OVX. However, the serum osteocalcin level in all the treatment groups decreased after 12 weeks treatment when comparing to the serum osteocalcin level before treatment: the OPG-Fc group decreased 35% (p < 0.05); ALN group decreased 23% (p < 0.05) while the OPG+ALN group decreased 58% (p < 0.05, Figure 3), however there was no statistical difference among all the treatment groups.

**Table 1:** Urinary calcium excretion (mmol/L) in different treatment groups over time

Treatment Groups	12 wks after OVX (before treatment)	24 wks after OVX (12 wks after treatment)
Sham	1.61 ± 0.30	1.81 ± 0.45

OVX	1.69 ± 0.25	2.11 ± 0.39 ^a^

OVX+ALN	1.67 ± 0.30	1.89 ± 0.33

OVX+OPG-Fc	1.56 ± 0.31	1.43 ± 0.35 ^b^

OVX+ALN+OPG-Fc	1.51 ± 0.40	1.48 ± 0.45 ^b^

(a): Group had higher value than that of all other groups at the given time, p < 0.05.

(b): Group had significantly lower value than that of Sham, OVX and OVX+ALN groups, p < 0.05

### Changes of Bone Mineral Density (BMD)

The BMD of L4-L6 lumbar vertebrae in all groups was similar before OVX and decreased about 20% at 12 weeks following OVX surgery in all groups compare to that of sham group. At 12 weeks after treatment (24 weeks after OVX surgery), the BMD of L4-L6 vertebrae in the OPG-Fc, ALN, OPG-Fc+ALN and sham control groups was significantly higher than that in the OVX group (Table 2), but there was no statistical difference among the OPG-Fc, ALN, OPG-Fc+ALN and sham control groups.

**Table 2:** Changes of BMD in L4-L6 vertebrae in different treatment groups over time

Groups	**L4-L6 Vertebrae Mean BMD (g/cm**^**2**^**)**		
	
	Before OVX	12 weeks post-OVX	24 weeks post-OVX
Sham	0.21 ± 0.046	0.34 ± 0.023	0.37 ± 0.017

OVX	0.22 ± 0.039	0.27 ± 0.039	0.27 ± 0.022**^a^**

OVX+ALN	0.21 ± 0.043	0.27 ± 0.039	0.37 ± 0.029

OVX+OPG-Fc	0.19 ± 0.038	0.23 ± 0.025	0.38 ± 0.026

OVX+ALN+OPG-Fc	0.19 ± 0.034	0.27 ± 0.020	0.36 ± 0.027

(a): Group had significantly lower value than that of all the other groups at the given time, p < 0.05.

### Mechanical Properties Measurements

At 24 weeks post OVX, the OVX group had significantly reduced the mechanical strength of L3 vertebrae, including ultimate load (-31%, Table 3) and ultimate stress (-41%, Table 3) compare to all other groups. The administration of ALN and OPG-Fc alone showed trends of minimizing loss of mechanical properties of vertebrae, but only the OPG-Fc +ALN group had achieved statistical significance compared to the OVX group (Table 3). The similar findings were seen in the mechanical properties of the femoral shaft: OVX reduced the ultimate load (-13%) and strength (-15%) significantly when compared to the sham group. At 24 weeks post-OVX, the values of ultimate force and ultimate strength were higher in the ALN, OPG-Fc and OPG-Fc+ALN groups than that in the OVX group, however only the OPG-Fc+ALN group achieved statistical significance (Table 3).

**Table 3:** Mechanical properties of L3 vertebra and femoral shaft at 24 weeks post-OVX

Groups (L3 vertebra)	Ultimate load (N)	Ultimate stress (N/mm^2^)
Sham	314.89 ± 22.87	46.30 ± 2.65

OVX	217.46 ± 33.04^a^	27.88 ± 4.23 ^a^

OVX+ALN	294.75 ± 25.58	37.79 ± 2.78

OVX+OPG-Fc	275.30 ± 28.77	35.29 ± 1.35

OVX+ALN+OPG-Fc	312.91 ± 41.76 ^b^	40.12 ± 2.53 ^b^

**Groups (Femoral shaft)**	**Ultimate load (N)**	**Ultimate stress (N/mm2)**

Sham	125.79 ± 11.19	187.14 ± 33.01

OVX	104.88 ± 6.21 ^a^	146.57 ± 12.75 ^a^

OVX+ALN	130.83 ± 9.11	179.91 ± 13.01

OVX+OPG-Fc	124.72 ± 6.78	174.65 ± 12.15

OVX+ALN+OPG-Fc	127.55 ± 7.49 ^b^	176.89 ± 17.44 ^b^

Data were presented as mean ± SEM.

(a): Group had significantly lower value than that of the sham group at the given time, p < 0.01.

(b): Group had significantly higher value than that of the OVX group at the given time, p < 0.01.

### Histomorphometric Measurements

Compared to the sham group, OVX group had a significant reduction in trabecular area, trabecular thickness, and increase in mineralizing surface and mineral apposition rate at 24 week post-OVX (Table 4). While the Tb.Ar% and trabecular thickness in the OVX+OPG-Fc, OVX+ALN and OVX+OPG-Fc+ALN groups were reached to the similar level or higher than that of sham and all were significantly higher than that in the OVX group at 24 weeks post-OVX; the OVX+OPG-Fc+ALN group had the highest Tb.Ar%, which was significantly higher (p < 0.05) than that of all other groups, including the sham group. OVX also increased the rate of bone turnover (BFR/BS) by 55% and osteoclasts number by 211% in the OVX group comparing to the sham group. For ALN-, OPG-Fc and OVX+OPG-Fc+ALN group, the mineral apposition rate, BFR/BS and osteoclasts number were significantly reduced than that in the OVX group at 24 weeks post-OVX. In the ALN+OPG-Fc group, the osteoclast number was lowest among all the groups and it was statistically significant when compared to all other groups, including the sham group. There was no significant difference of the mineralizing surface, mineral apposition rate and BRF/BS among the OPG-Fc, ALN and ALN+OPG-Fc groups (Table 4).

**Table 4:** Histomorphometric data at 24 weeks post-OVX in metaphyseal regions of the tibiae

Treatment Group	Tb.Ar (％)	Trabecular thickness (μm)	Min. surface (％)	Mineral apposition rate (μm/day)	BFR/BS (μm/day)	Osteoclast number (no/mm)
Sham	49.0 ± 9.23 ^a^	74.2 ± 11.34 ^a^	21.4 ± 3.3 ^a^	0.7 ± 0.13 ^a^	16.2 ± 1.45 ^a^	0.36 ± 0.03 ^a^

OVX	11.6 ± 5.81	45.2 ± 10.61	33.2 ± 4.6	1.2 ± 0.1	35.7 ± 2.30	1.12 ± 10.61

OVX+ALN	59.7 ± 8.29 ^a^	90.0 ± 17.65 ^a,b^	28.1 ± 1.44 ^a^	0.9 ± 0.0 ^a^	24.6 ± 1.11 ^a^	0.24 ± 0.04 ^a,b^

OVX+OPG-Fc	44.5 ± 6.72 ^a^	71.1 ± 27.19 ^a^	27.8 ± 2.17 ^a^	0.7 ± 0.0 ^a^	23.1 ± 2.11 ^a^	0.15 ± 0.04 ^a,b^

OVX+ALN+OPG-Fc	62.3 ± 9.34 ^a, b^	93.7 ± 13.97 ^a,b^	22.1 ± 3.71 ^a^	0.6 ± 0.09 ^a^	17.7 ± 1.94 ^a^	0.05 ± 0.00 ^a,b^

Data were presented as mean ± SEM.

(a): Group had significantly higher or lower value than that of the OVX group, p < 0.05.

(b): Group had significantly higher or lower value than that of the sham group, p < 0.05.

### Histology Examination

**Figure 4:**
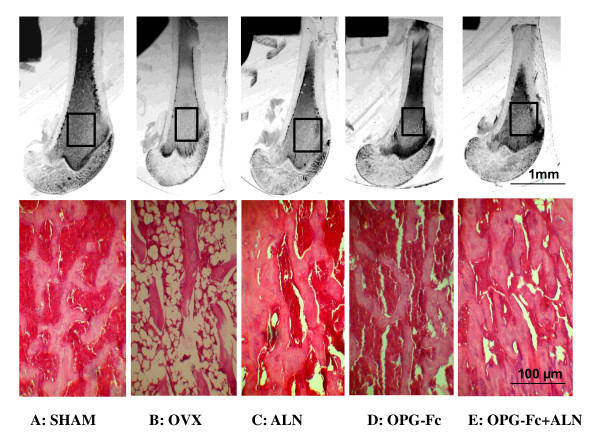
**Representatives of the distal tibiae macroscopic and histological appearances of different groups at 24 weeks post-OVX**. Top panel represents digital photographs of 200-μm thick sections (bar = 1 mm); bottom panel are representatives H&E histological sections from the boxed metaphyseal regions of the top panel (bar = 100 μm). The trabecular bone volume at the metaphyseal region of the tibiae in the OVX group had reduced markedly; both OPG-Fc and ALN-treated groups had greater bone volumes than that in the OVX group; and the OPG-Fc+ALN-treated group had the greatest bone volumes, which appeared to be even greater than that in the sham group.

On the microphotographs of the grounded sections of tibial metaphyseal regions, the trabecular bone volume was highest in the OPG-Fc+ALN group; followed by the Sham, OPG-Fc and ALN groups which were similar, and the OVX group had significant less bone volume than any of the groups (Figure 4, top panel). The histological sections of proximal tibial regions revealed that the trabecular volume and thickness were greatly reduced in the OVX group (Figure 4, bottom panel) compared to the sham group; whereas the trabecular volume and thickness in the ALN group and OPG group were similar to that of sham group and the OPG-Fc+ALN group had more trabecular volume than that of sham group (Figure 4, bottom panel).

## Discussion

Postmenopausal osteoporosis is a metabolic bone disease associated with estrogen deficiency and aging, having reduced bone mass that accounts for increased fracture risk. Antiresorptive agents have been developed and used clinically to suppress trabecular bone loss. OPG is a member of the tumour necrosis factor (TNF) receptor super-family, which negatively regulates osteoclastogenesis [8,9]. OPG inhibit osteoclast precursors differentiation into mature osteoclasts [10] and inhibits bone resorption in a dose-dependent manner [11]. RANKL is a member of the membrane-associated TNF ligand family that induces osteoclasts differentiation from the haemopoietic precursors and stimulates their bone resorptive activities [12,13]. OPG is a soluble decoy receptor for RANKL, it inhibits osteoclast differentiation and bone resorption via direct binding to OPG-expressed ligand secreted by osteoblasts or stromal cells [14]. The deficiency of OPG genes resulted in severe osteoporosis in both human and experimental animals [15,16], while systemic administration of rhOPG prevented osteopenia and ovariectomy-induced bone loss [1,14,15]. However, large dose of rhOPG is needed for a prolonged period when treating osteoporosis, the high cost of OPG and possible adverse immune reactions associated with the use of greater dose of OPG hinders the wider clinical application of OPG.

Alendronate (ALN, 4-amino-1-hydroxybutylidene bisphosphonate) is an amino bisphosphonate that has been developed for the treatment of osteolytic bone disorders such as giant cell tumor and osteoporosis. *In vivo*, alendronate has been localized at sites of bone resorption and inhibited osteoclastic activity, prevented and reversed the bone loss induced by estrogen deficiency, and maintained the mechanical strength of vertebrae in ovariectomized rats. The administration of ALN resulted in increased femoral cortical bending load as well as increased vertebral ultimate compressive load commensurate in a dose dependent manner. ALN has been found to lead to poor cell functioning or programmed cell death of osteoclasts, and the mechanism is through the inhibition of geranylgeraniol (a cell-permeable form of GGPP) and farnesol (a cell-permeable form of FPP) [17]. As a potent inhibitor of bone resorption, ALN has been proven to produce sustained reduction in biochemical markers of bone remodeling; while, consistent dose-related increases in bone mineral density in a variety of populations, especially elderly women [18].

We have used the OVX rat model which has been widely used to investigate estrogen-deficiency associated osteoporosis [19]. We have waited for 12 weeks following OVX to allow the development of osteopenic changes, and then the intervention treatments were given for 12 weeks to test the effect on preventing further bone loss.

It has been shown that administration of ALN (15 μg/kg) in the OVX rats had significantly increased bone mass and strength in OVX rats [20]. The dose of ALN used in this study was 28 μg/kg, which was nearly twice as much as the reported dose (15 μg/kg), and the animals tolerated well. The dose of OPG-Fc used in this study was 5 mg/kg, the same dose was reported by Capparelli et al. 2003 [21], whom showed that a single intravenous injection of rhOPG-Fc (5 mg/kg) in young growing rats causes significant gains in bone volume and density, which were associated with rapid and sustained suppression of osteoclastic bone resorption. We used rhOPG-Fc produced in yeast contains 180 residues from mature human OPG (amino acid 22-201) and 232 residues from the Fc protein of human IgG1, which has a longer circulating time due to its enhanced endothelial recycling and high molecular weight.

The main purpose of the current study was to test if the co-treatment of OPG-Fc with ALN will have any additive effect. In particular we were interested to see the effect of OPG-Fc in the animals which have already received ALN treatment as this has more clinical relevance for patients already on ALN treatment. The reasons we chose to use 2 weeks OPG-Fc treatment are that (1) Our pilot animal study has demonstrated that 5 mg/kg/day OPG-Fc injection for two weeks had anti-resporptive and anabolic effects in rats. (2) We have concerns of potential immunogenic reactions of rats to rhOPG-Fc when it was given for longer duration than 2 weeks. In the current study, we have administrated the rhOPG-Fc (5 mg/kg) by subcutaneous injection per day for 2 weeks, and the animals tolerated well and no adverse effect was observed.

The treatment of ALN, OPG-Fc and ALN+OPG-Fc all had anti-catabolic effects and prevented further bone loss in the OVX-rats. There was no statistical difference among the three treatment groups in terms of vertebrae BMD, mineralizing surfaces, mineral apposition rate, BFR/BS, suggesting that ALN and OPG-Fc treatment alone would be good enough to prevent bone loss in osteoporotic conditions. But only the ALN+OPG-Fc treatment group had significantly enhanced the vertebral anti-compressing strength and femoral shaft maximal loading for failure, suggesting the combined use of ALN and OPG-Fc could further enhance bone mechanical properties in addition to the bone mass. Since the mechanical testing is the current golden standard for accessing bone quality, the data suggested that the combined use of OPG-Fc and ALN in the current study not only prevented further bone loss (as the ALN and OPG-Fc treatment did), it also reversed bone quality following OVX-induced bone loss. The data also suggested that it may be beneficial for the patients who were already on ALN treatment to receive a short duration of OPG-Fc treatment, which may further enhance long bone mechanical properties.

We have showed RANKL protein expression in the vertebral bones was significantly decreased in the ALN and ALN+OPG-Fc treatment groups and OPG-Fc treatment for 2 weeks alone did not significantly affect RANKL protein expression. Since the OPG-Fc binds to RANKL directly to inhibit osteoclastogesis and it not necessarily has any effect on RANKL protein expression. We believe that the complex OPG-RANKL is still present under ALN treatment, but the RANKL expression was significantly decreased, so that additional OPG-Fc in the presence of ALN, even in a short duration (2 weeks) resulted in partial gain of bone mass and significantly improved bone structures, as indicated by the mechanical testing data.

One possible explanation on the greater anti-resorption effect of using OPG-Fc and ALN in combination is the amplified inhibitory effects of RNAKL function. rhOPG-Fc have competitive binding to RANKL whereas ALN leaded to a reduced expression of RANKL protein in the trabecular bone, thus the combination of the two treatments results a greater inhibitory effect on RANKL-dependent osteoclastogenesis and a positive balance towards bone formation cycle. ALN may have also inhibited farnesyl pyrophosphate synthesis or other enzymes of the mevalonate pathway which may lead to decrease GGPP biosynthesis and inhibit signal transduction in the Ras-MEK-ERK pathway, which is important in maintaining normal osteoclast function [22]. Small GTPases, such as Ras, Rho and Rac, are important for maintaining osteoclasts morphology and activity and lacking of these enzymes may lead to impaired function of osteoclasts [23-25]. Therefore, the combined use of OPG-Fc and ALN might have pushed the balance of bone remodeling cycle towards osteogenesis through amplified inhibition of osteoclastogenesis.

There are limitations of the current study, that the study duration was relatively short and we only tested one regime of rhOPG-Fc administration (by subcutaneous injection for 2 weeks) and the dose of rhOPG-Fc and ALN used in this study may not be the optimal one. Nonetheless, the current study serves as a proof-of-concept study and the exact mechanisms for how ALN reduce RANKL expression in bone; the optimal dosing and timing for ALN and OPG-Fc and the longer time effect of ALN+OPG-Fc on bone remodeling/formation needs future investigations.

In conclusion, we have demonstrated that co-treatment of ALN and OPG-Fc, through a short duration (2 weeks), has significantly improved the mechanical properties of femurs and vertebral bodies of the OVX rats compared to ALN and OPG-Fc single treatment groups. The combined use of rhOPG-Fc and ALN may be a new treatment strategy for preventing bone loss and reversing bone mass and quality in osteoprotic disorders, and it deserves further investigations.

## Competing interests

The authors declare that they have no competing interests.

## Authors' contributions

YW, PH and PFT carried out the animal experiments and participated in experimental design and the first draft of the manuscript. KMC helped with study design and discussion. GL and YW were involved in the study design and overall coordination, and YW was the grant holder. All authors read and approved the manuscript.
